# Imaging of the pulmonary vasculature in congenital heart disease without gadolinium contrast: Intraindividual comparison of a novel Compressed SENSE accelerated 3D modified REACT with 4D contrast-enhanced magnetic resonance angiography

**DOI:** 10.1186/s12968-019-0591-y

**Published:** 2020-01-23

**Authors:** Lenhard Pennig, Anton Wagner, Kilian Weiss, Simon Lennartz, Jan-Peter Grunz, David Maintz, Kai Roman Laukamp, Tilman Hickethier, Claas Philip Naehle, Alexander Christian Bunck, Jonas Doerner

**Affiliations:** 1 Institute for Diagnostic and Interventional Radiology, Faculty of Medicine and University Hospital Cologne, University of Cologne, Kerpener Straße 62, 50937 Cologne, Germany; 2 Philips GmbH, Hamburg, Germany; 3 Else Kröner Forschungskolleg Clonal Evolution in Cancer, University Hospital Cologne, Weyertal 115b, 50931 Cologne, Germany; 4 Department of Diagnostic and Interventional Radiology, University Hospital Würzburg, Oberdürrbacher Straße 6, 97080 Würzburg, Germany

**Keywords:** Magnetic resonance angiography, 4D CE-MRA, Contrast agent, Congenital heart disease, Pulmonary vasculature

## Abstract

**Background:**

Patients with Congenital heart disease (CHD) require repetitive imaging of the pulmonary vasculature throughout their life. In this study, we compared a novel Compressed SENSE accelerated (factor 9) electrocardiogram (ECG)- and respiratory-triggered 3D modified Relaxation-Enhanced Angiography without Contrast and Triggering (modified REACT-non-contrast-enhanced magnetic resonance angiography (modified REACT-non-CE-MRA)) with standard non-ECG-triggered time-resolved 4D CE-MRA for imaging of the pulmonary arteries and veins in patients with CHD.

**Methods:**

This retrospective analysis of 25 patients (June 2018–April 2019) with known or suspected CHD was independently conducted by two radiologists executing measurements on modified REACT-non-CE-MRA and 4D CE-MRA on seven dedicated points (inner edge): Main pulmonary artery (MPA), right and left pulmonary artery, right superior and inferior pulmonary vein, left superior (LSPV) and inferior pulmonary vein. Image quality for arteries and veins was evaluated on a four-point scale in consensus.

**Results:**

Twenty-three of the 25 included patients presented a CHD. There was a high interobserver agreement for both methods of imaging at the pulmonary arteries (ICC ≥ 0.96); at the pulmonary veins, modified REACT-non-CE-MRA showed a slightly higher agreement, pronounced at LSPV (ICC 0.946 vs. 0.895). Measurements in 4D CE-MRA showed higher diameter values compared to modified REACT-non-CE-MRA, at the pulmonary arteries reaching significant difference (e.g. MPA: mean 0.408 mm, *p* = 0.002). Modified REACT-non-CE-MRA (average acquisition time 07:01 ± 02:44 min) showed significant better image quality than 4D CE-MRA at the pulmonary arteries (3.84 vs. 3.32, *p* < 0.001) and veins (3.32 vs. 2.72, *p* = 0.015).

**Conclusions:**

Compressed SENSE accelerated (factor 9) ECG- and respiratory-triggered 3D modified REACT-non-CE-MRA allows for reliable and fast imaging of the pulmonary arteries and veins with higher image quality and slightly higher interobserver agreement than 4D CE-MRA without contrast agent and associated disadvantages. Therefore, it represents a clinically suitable technique for patients requiring repetitive imaging of the pulmonary vasculature, e.g. patients with CHD.

## Background

With an incidence of 6–8/1000 at birth, congenital heart disease (CHD) comprises a wide range of different manifestations regarding the cardiovascular system potentially leading to death if left untreated. CHD has shown a serious improvement of survival over the past decades due to advancement in surgical techniques and early diagnosis, mostly owing to the widely use of fetal echocardiography [1–3]. Echocardiography represents the primary imaging modality of choice as it allows for fast, accurate and non-invasive imaging of cardiac function and vessel morphology [3–5]. However, it suffers from limitations such as user dependency and limited field of view (FOV) in growing patients [6, 7].

Given the radiation dose as well as the use of iodinated contrast agent in computed tomography (CT)-angiography (CTA) and digital subtraction angiography (DSA) with the invasiveness of the latter, cardiovascular magnetic resonance (CMR) has been established as the non-invasive imaging of choice to evaluate the different vascular territories of the thorax in patients with CHD and has to be regarded as the gold standard [6, 8–10]. Besides 4D flow CMR, contrast-enhanced MR-angiography (CE-MRA) has proven to sufficiently detect vascular abnormalities and has shown technical progress over the past decades with the development of time-resolved 4D CE-MRA [8, 11–13]. However, the accurate application of CE-MRA and 4D CE-MRA is technically demanding and shows further limitations such as nephrogenic systemic fibrosis (NSF) [14] and long term retention of gadolinium.

Therefore, many non-CE-MRA techniques have been developed in the past, including sequences based on turbo spin echo (TSE), spoiled gradient echo sequences, steady-state free precession (SSFP), and balanced SSFP (bSSFP) with SSFP and bSSFP being the most widely used for the assessment of the pulmonary vasculature in patients with CHD as well as other diseases affecting the pulmonary vessels, e.g. pulmonary hypertension (PH) [15–19]. Recently, a novel 3D Relaxation-Enhanced Angiography without Contrast and Triggering (REACT) sequence, a combination of non-volume-selective short tau inversion recovery (STIR) pulse, a T2 preparation (T2 prep) pulse, and dual gradient echo Dixon (mDIXON XD), was introduced. It combines the benefits of SSFP with robust fat and background suppression [20].

The purpose of this study was to investigate the feasibility of a novel electrocardiogram (ECG)- and navigator-triggered 3D non-CE-MRA based on a modified REACT approach (modified REACT-non-CE-MRA) for the imaging of the pulmonary arteries and veins in patients with CHD and to compare measurement values and image quality to standard 4D CE-MRA.

## Methods

### Patient population

Patients were retrospectively selected from our internal database of 26 consecutive patients over a ten-month study period (June 2018–April 2019) receiving a dedicated clinical protocol regarding known or suspected CHD including both, 4D CE-MRA and modified REACT-non-CE-MRA. Insufficient contrast in 4D CE-MRA led to exclusion of patients. There were no exclusions regarding pathologies or operative treatment. Due to the retrospective design of the study, the local ethics committee waived written informed consent requirement in the patient cohort.

### Image acquisition

All scans were performed on a clinical whole body 1.5 T CMR system (Philips Ingenia, Philips Healthcare, Best, The Netherlands) equipped with a dedicated 28-channel coil for cardiac imaging. The protocol comprised a non-CE-MRA using a modified REACT approach, a 4D CE-MRA, and 2D bSSFP breath hold cine imaging in standard orientations (4-chamber, 2-chamber, 3-chamber, short axis, transversal, left ventricular outflow tract, right ventricular outflow tract (RVOT)) as well as phase contrast velocity measurements of the main pulmonary artery (MPA) and the ascending aorta.

For 4D CE-MRA, a 3D spoiled gradient echo sequence was used. Gadobutrol (Gadovist, Bayer HealthCare Pharmaceuticals, Berlin, Germany; 0.1 ml/kg body weight) was injected at a flow-rate of 2 ml/second into an antecubital vein. Patients were asked to perform a breath hold during the acquisition. To allow for high spatio-temporal resolution, the acquisition was combined with parallel imaging using SENSitivity Encoding (SENSE) and a keyhole technique where 20% of the central k-space was acquired in each dynamic (4D Track, Philips Healthcare). The keyhole data was then combined with outer k-space data from a reference scan during image reconstruction.

For non-CE-MRA, imaging was based on a modified flow-independent REACT sequence. 3D magnetization-prepared mDIXON XD (Philips Healthcare) was combined with a 30 ms T2 prep sequence. Since it was verifiable that background suppression of mDIXON XD in combination with T2 prep was sufficient for cardiovascular applications, no STIR preparation was applied, contrary to the original REACT sequence as introduced by Yoneyama et al. [20]. To compensate for cardiac and respiratory motion, ECG-triggering (end-diastolic) and respiratory navigator-triggering were added to the originally proposed REACT sequence, therefore being referred to as “modified” REACT-non-CE-MRA throughout the manuscript. Data were acquired in the coronal plane. For acceleration of image acquisition, Compressed SENSE (Philips Healthcare), a combination of compressed sensing and parallel imaging using SENSE, was used [21, 22]. For data acquisition, a variable density incoherent sampling pattern with high-density sampling in the center and continuously increased undersampling towards the k-space periphery was employed. Image reconstruction was based on an iterative L1 norm minimization, assuring data consistency and image sparsity in the wavelet domain. Additionally, the reconstruction was regularized by coil sensitivity distribution and SENSE parallel imaging. Reconstruction was done online on the standard hardware as provided by the manufacturer of the CMR system. An acceleration factor of 9 was used, resulting in a nominal scan time of 02:11 min.

Detailed imaging parameters are given in Table 1.

**Table 1:** Imaging parameters of modified REACT-non-CE-MRA and 4D CE-MRA. FOV = field of view. TR = repetition time. TE = echo time

	REACT-non-CE-MRA	4D CE-MRA
Acquisition matrix	235 × 299 × 100	268 × 268 × 25
Resolution [mm]	1.7 × 1.7 × 1.7	1.5 × 1.5 × 4
FOV [mm]	400 × 508 × 170	400 × 400 × 100
Flip Angle [deg]	10	30
TR/TE1/TE2 [ms]	6.3/1.8/4	2.8/1.05
T2 preperation [ms]	30	–
k-space lines per heartbeat	35	–
Acceleration factor	Compressed SENSE 9	SENSE 3
Temporal resolution	–	1 s
Nominal scan time [min]	02:11	~ 0:24
Subtraction	–	CE – native

### Measurement

Anonymized images of 4D CE-MRA and modified REACT-non-CE-MRA were presented in random order to two radiologists (L.P., A.W.), each with at least two years of experience in cardiovascular imaging, who independently conducted the measurement on seven distinct measurement points:

1. MPA (2 cm distal of the pulmonary valve as correlated by RVOT/transversal cine if necessary).
2. Right pulmonary artery (RPA, 1 cm distal of the bifurcation).
3. Left pulmonary artery (LPA, 1 cm distal of the bifurcation).
4. Right superior pulmonary vein (RSPV, 1 cm proximal of the ostium).
5. Right inferior pulmonary vein (RIPV, 1 cm proximal of the ostium).
6. Left superior pulmonary vein (LSPV, 1 cm proximal of the ostium).
7. Left inferior pulmonary vein (LIPV, 1 cm proximal of the ostium).

For each point, the measurement (inner diameter approach) was conducted on source images using the manual Multiplanar-Reconstruction-(MPR) tool in IMPAX EE (Agfa Healthcare N.V., Mortsel, Belgium) in manual perpendicular alignment. Maximum intensity projection images were not used since they lead to an apparent reduction in vessel diameter resulting in an underestimation [23]. Measurement points were excluded when they could not be assessed due to susceptibility or severe pulsation artifacts.

### Image quality evaluation

Image quality was evaluated by both observers in consensus on a four-point scale regarding sharpness, presence of pulsation artifacts at the levels of measurement, and anatomic delineation. Quality was rated on a Likert scale of 1 to 4: 1 non-diagnostic, 2 poor image quality with substantial blurring impairing diagnostic confidence, 3 intermediate image quality with mild blurring, and 4 good image quality without any blurring and resulting high diagnostic confidence.

### Statistical analysis

Data are shown as mean ± standard deviation (SD), unless noted otherwise. Statistical significance was set at *p* < 0.05. For each point of measurement, the average diameter of two tangential measurements was used for analysis. To evaluate interobserver reliability, intraclass correlation coefficients (ICCs) were calculated. Bland-Altman analysis was conducted to assess differences regarding measurement values of the pulmonary arteries and veins using 4D CE-MRA and modified REACT-non-CE-MRA. A paired t-test was used to evaluate the significance of differences between pulmonary artery and vein measurements on both modalities. For evaluation of differences regarding image quality, the Wilcoxon matched pair test was applied. Statistical analysis and graph creation were performed using JMP (Version 14.1.0, SAS Institute, Cary, North Carolina, USA).

## Results

### Study population and baseline characteristics

Of the 26 patients, one patient was excluded due to insufficient contrast in 4D CE-MRA, resulting in a study population of 25 patients (39 ± 20 years, body mass index 23.8 ± 5.2; 15 male subjects). Twenty-three patients presented a CHD, the most frequent being an atrial septal defect (*n* = 7), followed by ventricular septal defect (*n* = 5), transposition of the great arteries (*n* = 3), tetralogy of Fallot (TOF, *n* = 2) and pulmonary atresia (*n* = 2). 10 patients had surgery for CHD prior to the examination.

### Imaging

All included imaging studies were executed without any complications. Modified REACT-non-CE-MRA showed an average total acquisition time of 7:01 ± 2:44 min (depending on the patient’s breathing frequency as well as heart rate), 4D CE-MRA of 2:14 ± 1:01 min.

### Interobserver agreement of modified REACT-non-CE-MRA and 4D CE-MRA

At pulmonary arteries, modified REACT-non-CE-MRA and 4D CE-MRA showed comparable ICCs between 0.95 and 0.99. At pulmonary veins, modified REACT-non-CE-MRA achieved a higher agreement than 4D CE-MRA with the highest difference at LSPV (0.95 versus 0.90). Detailed results are given in Table 2.

**Table 2:** Interobserver correlation coefficients of both methods of imaging and the dedicated measurement points with values > 0.8 indicating excellent correlation

	MPA	RPA	LPA	RSPV	RIPV	LSPV	LIPV
Modified REACT-non-CE-MRA	0.9904	0.9866	0.9792	0.935	0.959	0.9457	0.9595
4D CE-MRA	0.9778	0.9834	0.9594	0.9101	0.9478	0.895	0.9347

MPA = main pulmonary artery. RPA = right pulmonary artery. LPA = left pulmonary artery. RSPV = right superior pulmonary vein. RIPV = right inferior pulmonary vein. LSPV = left superior pulmonary vein. LIPV = left inferior pulmonary vein

### Detailed comparison between 4D CE-MRA and modified REACT-non-CE-MRA

**Fig. 1:**
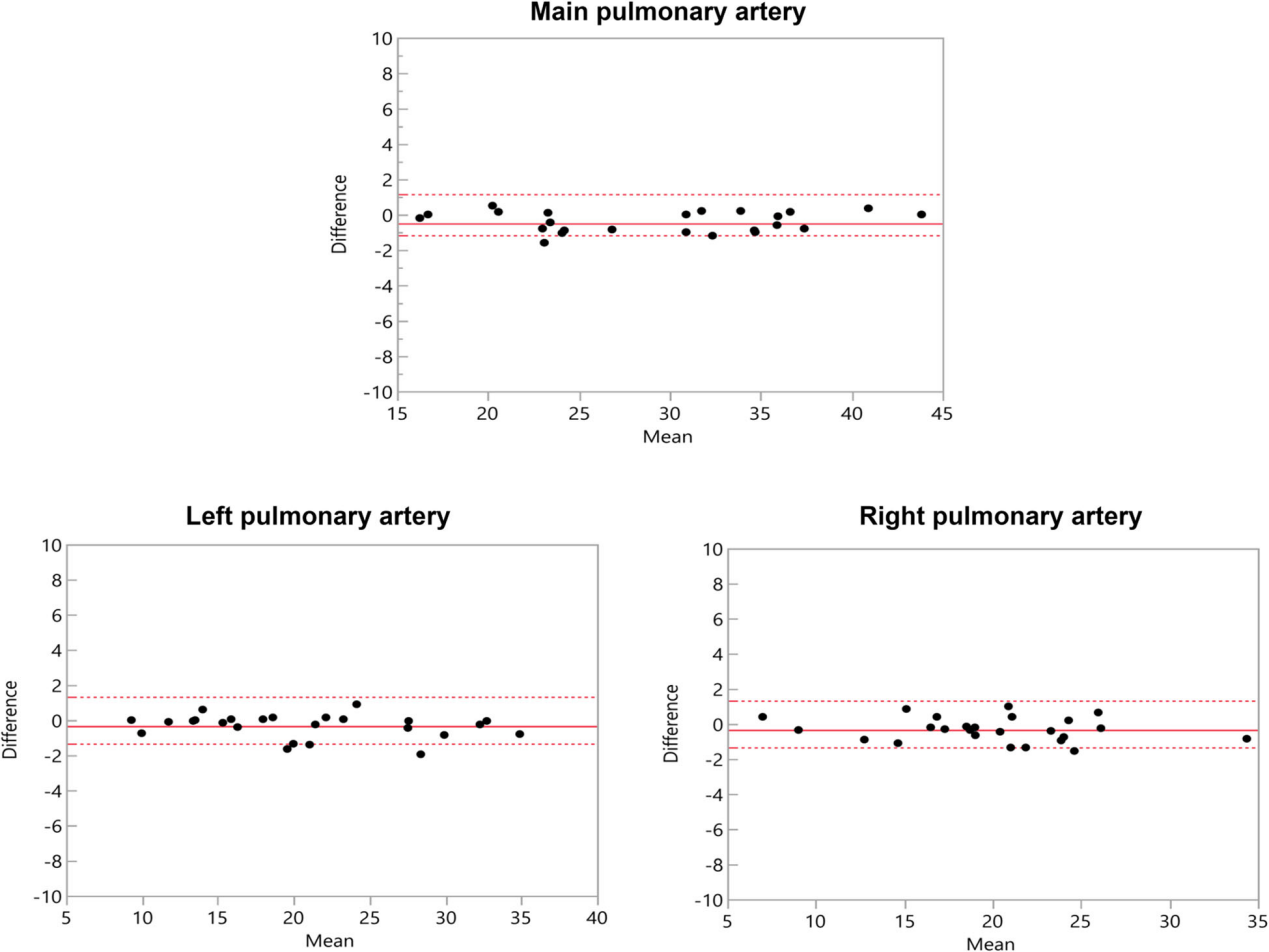
Bland–Altman comparison of the measured diameters of the pulmonary arteries assessed by modified REACT-non-CE-MRA and 4D CE-MRA. The middle line indicates the mean bias of the diameter measurements whereas the dotted lines represent the 95% confidence interval. Values are given in mm

**Fig. 2:**
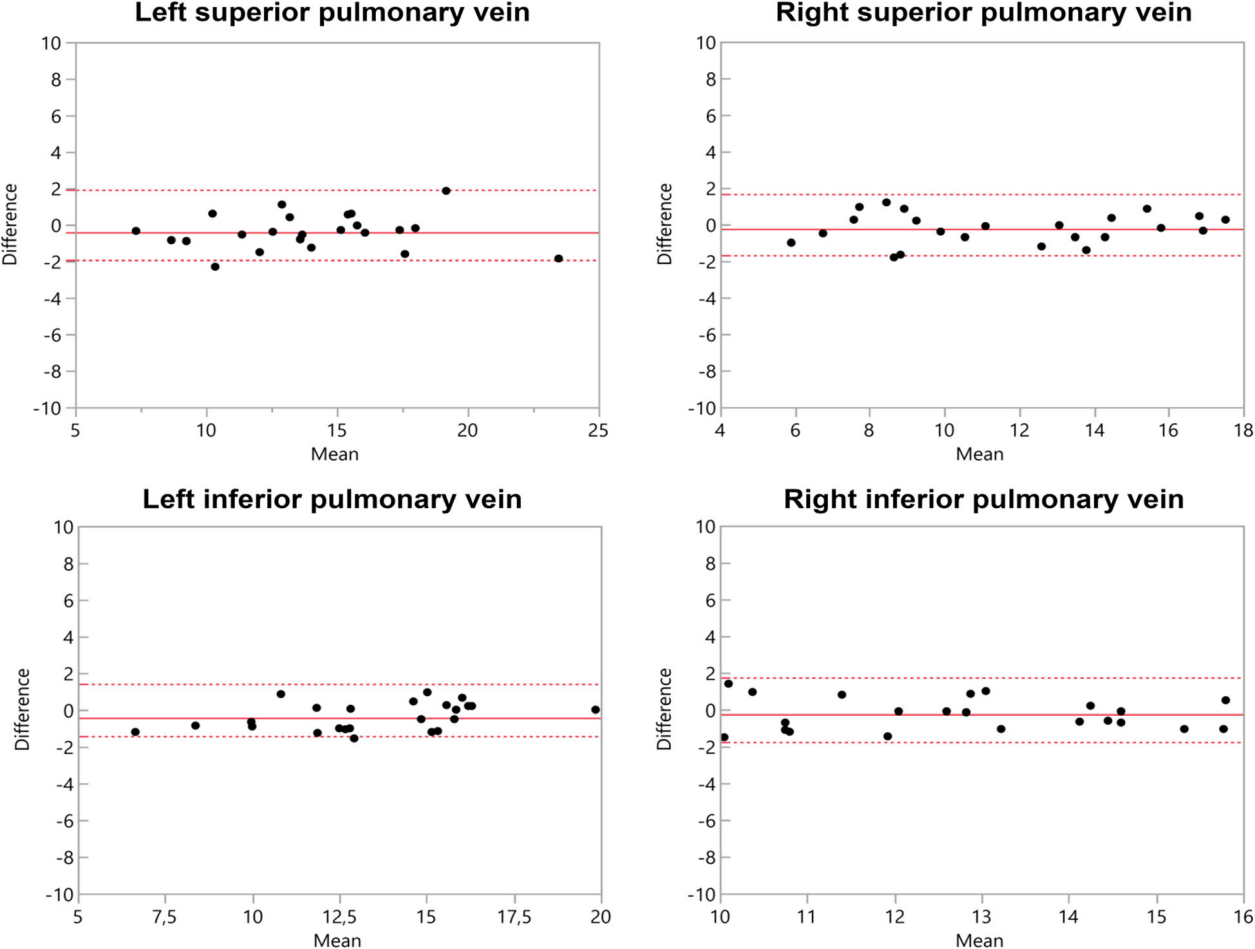
Bland–Altman comparison of the measured diameters of the pulmonary veins assessed by modified REACT-non-CE-MRA and 4D CE-MRA. The middle line indicates the mean bias of the diameter measurements whereas the dotted lines represent the 95% confidence interval. Values are given in mm

4D CE-MRA showed greater diameters at all points of measurement with significant differences at MPA (*p* = 0.002), RPA (*p* = 0.019), LPA (*p* = 0.026) (Table 3). At the pulmonary veins, no significant difference was noted with RSPV yielding the highest difference (0.396 mm) (Table 3). Bland-Altman comparisons of the differences regarding measurement values of the pulmonary arteries and veins assessed by 4D CE-MRA and modified REACT-non-CE-MRA with corresponding 95% confidence intervals are given in Figs. 1 and 2. Due to impaired image quality, measurement was not possible at the LSPV in one patient and at the LIPV in three patients using 4D CE-MRA. In modified REACT-non-CE-MRA, all measurements were conducted sufficiently.

**Table 3:** Average measurement diameters and differences as well as the results of the paired t-test between differences of both methods of imaging at the dedicated measurement points, bold indicating statistical significance (*p* < 0.05)

	MPA	RPA	LPA	RSPV	RIPV	LSPV	LIPV
Modified REACT non-CE MRA, mean, diameter, mm, SD	29.0 ± 7.5	20.7 ± 7.1	19.7 ± 5.6	13.8 ± 3.7	13.3 ± 3.1	11.5 ± 3.4	12.7 ± 2.0
4D CE-MRA, mean, diameter, mm, SD	29.4 ± 7.5	21.0 ± 7.3	20.0 ± 5.7	14.2 ± 3.7	13.7 ± 3.0	11.8 ± 3.5	13.0 ± 2.0
Differences, mean, mm	0.4	0.3	0.3	0.4	0.4	0.2	0.3
95% confidence interval, cm	0.2 to 0.7	0.1 to 0.6	0.0 to 0.6	0.0 to 0.9	0.1 to 0.7	−0.1 to 0.6	− 0.1 to 0.7
*p* value	**0.002**	**0.019**	**0.026**	0.0638	0.0199	0.192	0.173

MPA = main pulmonary artery. RPA = right pulmonary artery. LPA = left pulmonary artery. RSPV = right superior pulmonary vein. RIPV = right inferior pulmonary vein. LSPV = left superior pulmonary vein. LIPV = left inferior pulmonary vein

### Comparison of image quality between 4D CE-MRA and modified REACT-non-CE-MRA

Modified REACT-non-CE-MRA showed average image quality scores of 3.8 for pulmonary arteries and 3.3 for veins compared to values of 3.3 for arteries (*p* < 0.001) and 2.7 for veins (*p* = 0.015) in 4D CE-MRA.

**Fig. 3:**
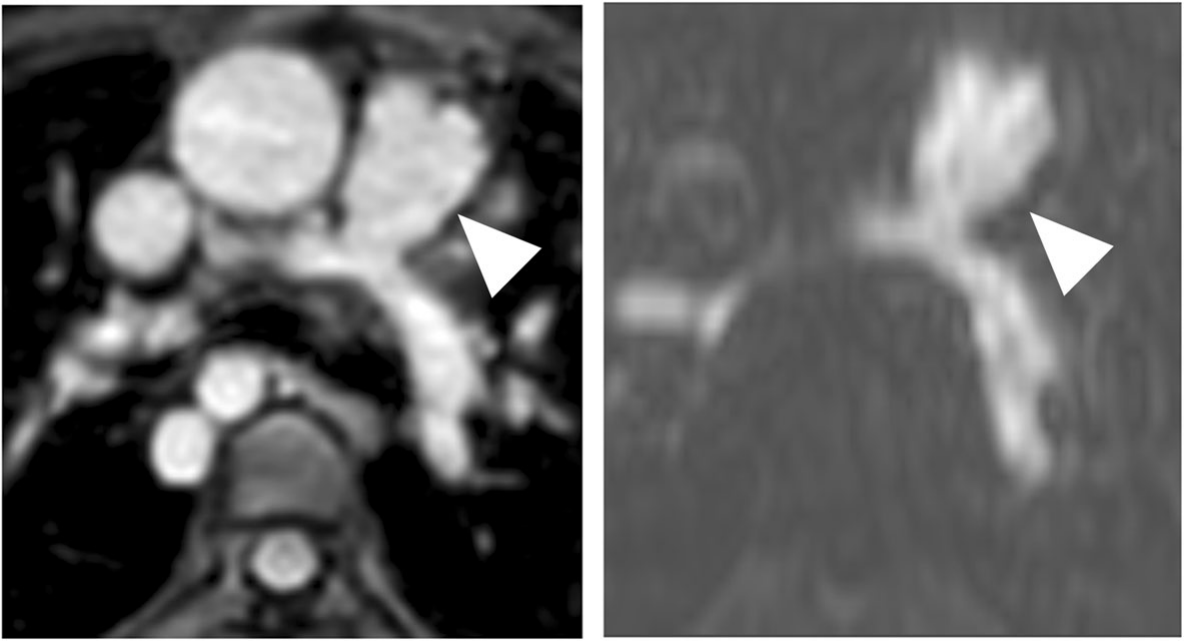
Multiplanar reformatted image of the main pulmonary artery (arrowheads) in an 11-year-old patient with pulmonary atresia after implantation of a Contegra conduit and multiple angioplasties of both pulmonary arteries. The pulmonary arteries can be clearly delineated in modified REACT-non-CE-MRA (left) compared to the blurred appearance in 4D CE-MRA (right)

**Fig. 4:**
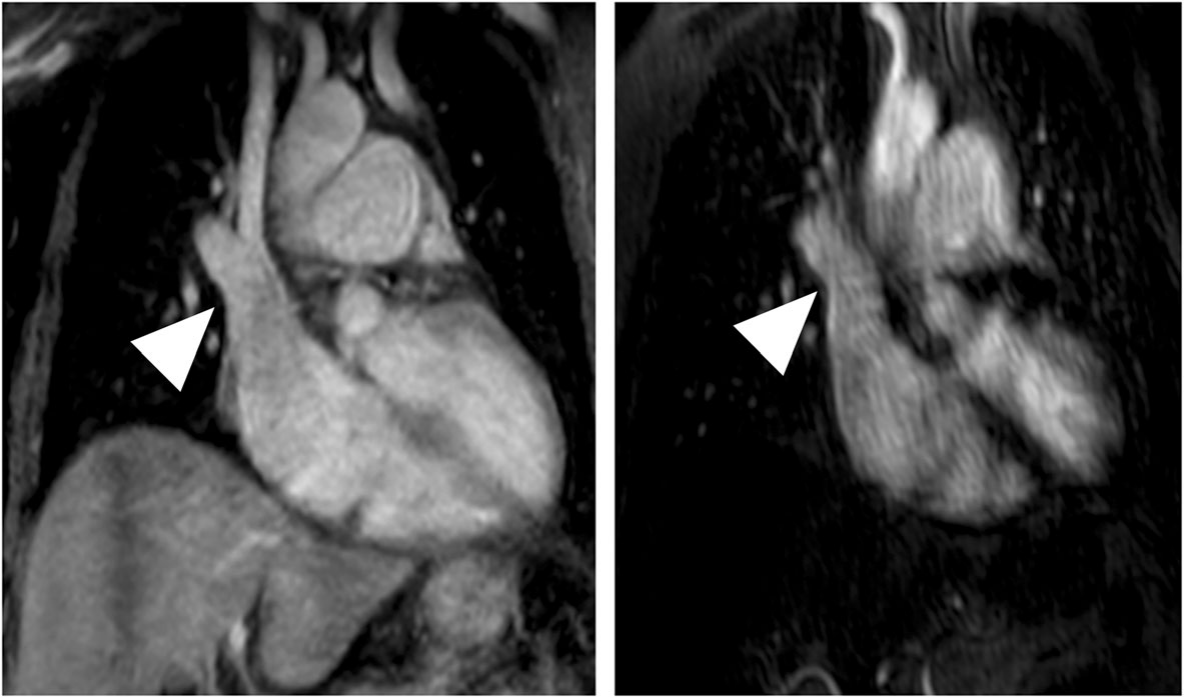
CMR-imaging in a 56-year-old patient with sinus venosus atrial septal defect and suspected associated anomalous pulmonary venous return. Modified REACT-non-CE-MRA (left) clearly depicts connection of the right superior pulmonary vein with the superior vena cava (arrowheads) whereas 4D CE-MRA (right) shows pulsation artefacts hampering diagnosis

**Fig. 5:**
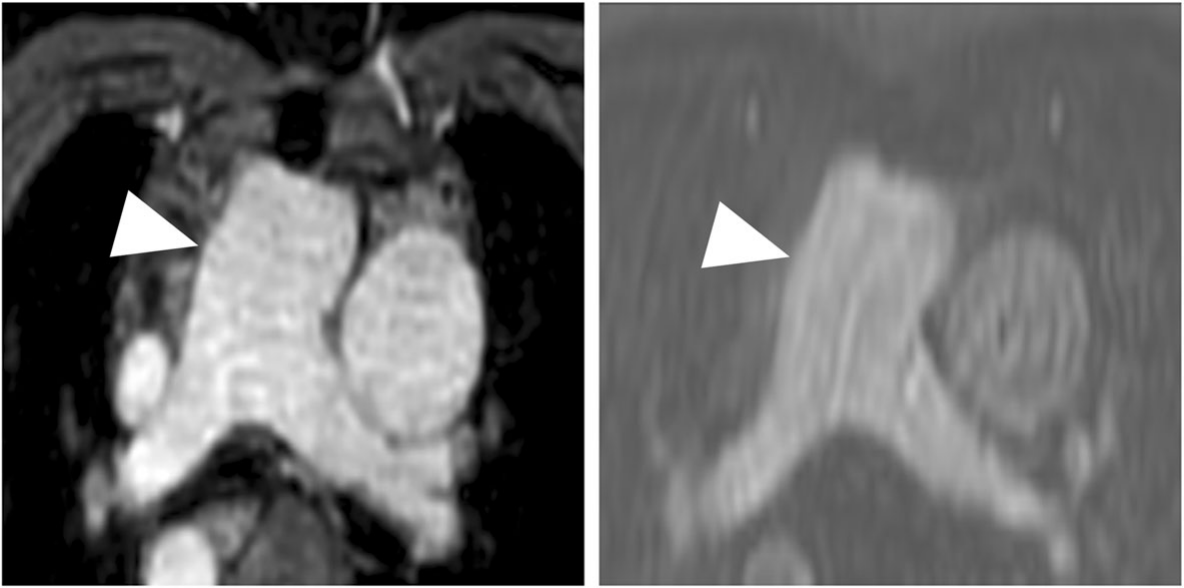
Multiplanar reformatted image of the main pulmonary artery (arrowheads) in a 39-year-old patient with situs inversus totalis and dextrocardia with transposition of the great artery and arterial switch operation (left: modified REACT-non-CE-MRA, right: 4D CE-MRA). Modified REACT-non-CE-MRA shows superior delineation of the vessel wall compared to 4D CE-MRA due to pulsation artifacts

**Fig. 6:**
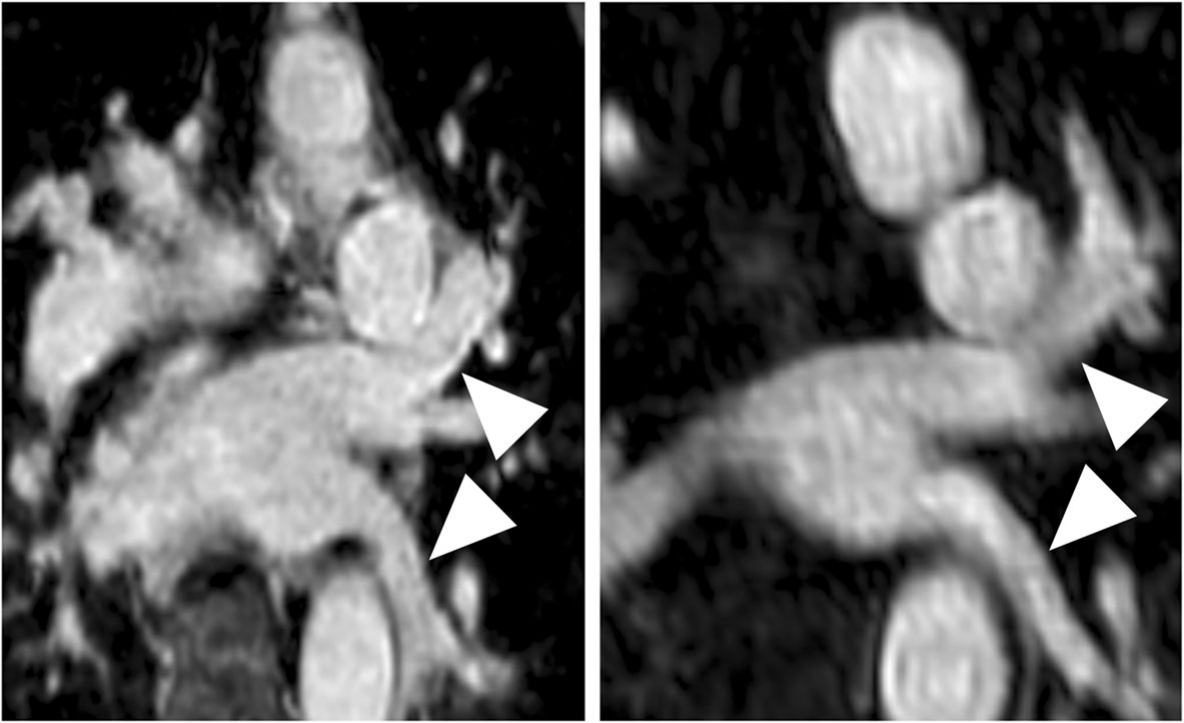
Multiplanar reformatted image of the left pulmonary veins (arrowheads; left: modified REACT-non-CE-MRA, right: 4D CE-MRA) in a 71-year-old patient with patent ductus arteriosus. Modified REACT-non-CE-MRA shows improved delineation of the vessel wall compared to 4D CE-MRA

Figures 3, 4, 5 and 6 give exemplary comparisons of modified REACT-non-CE-MRA and 4D CE-MRA.

## Discussion

In our study, we retrospectively compared a novel Compressed SENSE accelerated navigator- and ECG-triggered 3D modified REACT-non-CE-MRA with standard non-ECG-triggered 4D CE-MRA for the imaging of the pulmonary vessels in patients with CHD. The major findings of this study are the following: 1. 4D CE-MRA showed greater diameters for the pulmonary vessels in comparison to modified REACT-non-CE-MRA, at the arteries with significant difference. 2. Modified REACT-non-CE-MRA offers a significantly higher image quality of the pulmonary vasculature and a slightly higher interobserver agreement at the pulmonary veins than 4D CE-MRA.

In line with previous works, which compared non-ECG-triggered CE-MRA with ECG-triggered non-CE-MRA (SSFP, end-diastolic) for the imaging of pulmonary arteries and veins, 4D CE-MRA showed higher measurement values of the pulmonary vessels compared to modified REACT-non-CE-MRA, at the arteries with a significant difference [16, 24]. These differences are mainly due to pulsation and breathing artifacts in 4D CE-MRA and the resulting hampered vessel delineation. These pulsation artifacts are pronounced in patients with CHD such as TOF who show highly pulsatile circulations of the pulmonary arteries [23]. Furthermore, the motion and the changing size of the pulmonary veins and arteries throughout the cardiac cycle have to be considered when comparing the non-triggered acquisition of 4D CE-MRA with the ECG-triggered (end-diastolic) acquisition of modified REACT-non-CE-MRA, hence resulting in physiological differences which lead to greater diameters in 4D CE-MRA [16, 23–26]. Furthermore, the above-mentioned factors also implicate the slightly higher interobserver agreement of modified REACT-non-CE-MRA at the pulmonary veins. In line with above-mentioned studies, which have shown that other ECG-triggered and respiratory-gated non-CE-MRA sequences such as 3D SSFP outperform untriggered CE-MRA in terms of image quality, modified REACT-non-CE-MRA with respective triggering provided significant higher image quality scores of the pulmonary arteries and veins than 4D CE-MRA [15, 16, 18].

Patients with CHD require repetitive imaging of the pulmonary vasculature throughout their life and CMR has to be regarded as the gold standard of imaging with CE-MRA being used to evaluate the different vascular territories of the thorax and to detect its pathologies [6, 8–10]. However, the accurate execution of CE-MRA and 4D CE-MRA is technically demanding, as image quality depends on good coordination among the contrast injection, exact timing of data acquisition and patient cooperation for breath holding, subsequently limiting its use in incompliant patients [9, 16]. Recently, 4D CE-MRA has been developed, which offers the possibility to acquire a series of volume angiograms in quick succession therefore simplifying the timing of acquisition in relation to the passage of the contrast bolus [9]. Nevertheless, the majority of time-resolved MRA uses some form of data sharing across time, making them sensitive to respiratory motion artifacts; therefore the majority is performed during a breath-hold. New techniques of free-breathing time-resolved MRAs are technically feasible, but show decreased signal- and contrast-to-noise ratios [9].

Furthermore, the use of gadolinium contrast poses a drawback potentially leading to NSF in end-stage renal disease, anaphylactic reactions or extravasation. Contrast agents show high costs and require an intravenous access, potentially limiting its use in the clinical routine [14, 27–30]. Given the growing literature on long-term gadolinium deposition within the brain and the uncertainty regarding its long-term effects, repetitive application of gadolinium contrast, especially in children, should be executed cautiously [31–33].

Consequently, many non-CE-MRA methods have been developed over the past decades with SSFP and bSSFP being routinely used for thoracoabdominal vessels. 3D bSSFP/SSFP show advantages such as high signal-to-noise ratios, high blood-to-tissue contrast due to its bright-blood signal and flow independence [34–37]. However, they are highly sensitive to off-resonance effects caused by B0 heterogeneities in the main magnetic field and disruptions of the steady state due to highly pulsatile flow or motion and show high background signals [15, 38, 39]. Therefore, image quality can be impaired by signal loss, banding artifacts and insufficient fat suppression. These effects are pronounced in higher magnetic fields such as 3 T and in large FOVs. When applied in large FOVs, a long acquisition time is required, consequently limiting its use in clinical routine [15, 37].

Recently, a Compressed SENSE accelerated 3D REACT-non-CE-MRA was introduced, which overcomes these limitations by the following: On the one hand, mDIXON XD combines the above-mentioned benefits of SSFP with reduced sensitivity to inhomogeneities in the magnetic field [20, 40]. mDIXON XD provides robust suppression of fat and background and allows for separation of water and fat, consequently leading to insensitivity of REACT-non-CE-MRA to inhomogeneities in the magnetic field, even in large FOVs [41]. Therefore, it provides high-resolution scans in large FOVs and allows the application in higher magnetic fields such as 3 T, where inhomogeneities are expected to be higher. On the other hand, with the advent of new acceleration techniques such as compressed sensing, shorter acquisition times beyond current parallel imaging techniques are possible, especially when combining the advantages of both techniques [22, 42]. In this work, Compressed SENSE was employed, allowing for image-acquisition acceleration factors currently not achievable by compressed sensing or parallel imaging alone. Consequently, this directly addresses shortcomings of non-CE-MRA techniques as identified in previous work [15, 36]. The Compressed SENSE technique was fully integrated on the clinical system, resulting in reconstruction times below one minute and an overall scan time of 7:01 ± 2:44 min, lower than 3D SSFP for the same kind of investigation and FOV (10:01 ± 4.5 min) [15]. REACT-non-CE-MRA was combined with respiratory navigator-triggering and ECG-triggering to compensate for respiratory and cardiac motion, therefore being referred to as “modified” REACT-non-CE-MRA in this study. Given its flow-independency, REACT-non-CE-MRA can in principle also be used without triggering, making it a versatile alternative to CE-MRA, especially for patients who are unable to perform a breath-hold, e.g. children.

Modified REACT-non-CE-MRA only delivers a stationary depiction of the vessels and does not include dynamic flow conditions as 4D CE-MRA [8]. Given the fact that modified REACT-non-CE-MRA enables a simultaneous display of arterial and venous vessels of the thorax unlike 4D CE-MRA, resulting images might appear overloaded. However, since the vessels are displayed sharply and in good quality, a sufficient differentiation is possible.

### Clinical applications

Besides the depiction of the pulmonary vasculature in CHD, modified REACT-non-CE-MRA may offer an alternative to CTA or other MRA techniques for patients with PH or suspected pulmonary embolism as well as for imaging of the aorta in patients with connective tissue disease. Further, its use in other vascular territories, e.g. the extracranial arteries, might pose an additional clinical application and may warrant future investigation.

### Limitations

The size and the heterogeneity of the study population with its wide range of different CHDs and postoperative alterations may be regarded as a drawback of this study.

We did not compare modified REACT-non-CE-MRA to DSA and regarded untriggered breath-hold 4D CE-MRA as the reference standard. Furthermore, no comparison to other ECG-triggered and respiratory-gated non-CE-MRA sequences such as 3D SSFP was conducted in this study, which may nurture future investigations.

Additionally, the lower resolution of 4D CE-MRA consequently leads to an inferior image quality than modified REACT-non-CE-MRA and offers lower image quality than standard CE-MRA [8]. The comparison of the ECG- and navigator-triggered modified REACT-non-CE-MRA with an untriggered breath-hold first-pass CE-MRA (4D CE-MRA) may represent a limitation of this work given the fact that ECG-gating as well as navigator-gating with slow-infusion of contrast media have proven to increase the image quality of CE-MRA [43–47]. However, ECG-gating with respect to contrast bolus arrival proves to be challenging in daily clinical routine.

## Conclusions

Compressed SENSE accelerated (factor 9) ECG- and respiratory navigator-triggered 3D modified REACT-non-CE-MRA allows for robust and reliable imaging of the pulmonary vasculature in CHD with a higher image quality and a slightly higher interobserver agreement than 4D CE-MRA without the need of gadolinium contrast. Given its short acquisition time, it represents a clinically applicable alternative for patients with CHD in need of repetitive imaging of the pulmonary vessels.

## Data Availability

The datasets generated and/or analysed during the current study are not publicly available due to data protection but are available from the corresponding author on reasonable request.

## Abbreviations

bSSFP: Balanced SSFP
CE-MRA: Contrast-enhanced MR-angiography
CHD: Congenital heart disease
CMR: Cardiovascular magnetic resonance
CT: Computed tomography
CTA: CT-angiography
DSA: Digital subtraction angiography
ECG: Electrocardiogram
FOV: Field of view
ICC: Intraclass correlation coefficients
LIPV: Left inferior pulmonary vein
LPA: Left pulmonary artery
LSPV: Left superior pulmonary vein
mDIXON XD: Dual gradient echo Dixon
MPA: Main pulmonary artery
MPR: Multiplanar reconstruction
NSF: Nephrogenic systemic fibrosis
PH: Pulmonary hypertension
REACT: Relaxation-Enhanced Angiography without Contrast and Triggering
RIPV: Right inferior pulmonary vein
RPA: Right pulmonary artery
RSPV: Right superior pulmonary vein
RVOT: Right ventricular outflow tract
SD: Standard deviation
SENSE: SENSitivity encoding
SSFP: Steady-state free precession
STIR: Short tau inversion recovery
T2 prep: T2 preparation
TOF: Tetralogy of Fallot
TSE: Turbo spin echo
