# Correction to: Imaging of the pulmonary vasculature in congenital heart disease without gadolinium contrast: Intraindividual comparison of a novel Compressed SENSE accelerated 3D modified REACT with 4D contrast-enhanced magnetic resonance angiography

**DOI:** 10.1186/s12968-020-00606-2

**Published:** 2020-02-21

**Authors:** Lenhard Pennig, Anton Wagner, Kilian Weiss, Simon Lennartz, Jan-Peter Grunz, David Maintz, Kai Roman Laukamp, Tilman Hickethier, Claas Philip Naehle, Alexander Christian Bunck, Jonas Doerner

**Affiliations:** 1 Institute for Diagnostic and Interventional Radiology, Faculty of Medicine and University Hospital Cologne, University of Cologne, Kerpener Straße 62, 50937 Cologne, Germany; 2 Philips GmbH, Hamburg, Germany; 3 Else Kröner Forschungskolleg Clonal Evolution in Cancer, University Hospital Cologne, Weyertal 115b, 50931 Cologne, Germany; 4 Department of Diagnostic and Interventional Radiology, University Hospital Würzburg, Oberdürrbacher Straße 6, 97080 Würzburg, Germany

**Correction to: J Cardiovasc Magn Reson**

**https://doi.org/10.1186/s12968-019-0591-y**

The original publication of this article [1] contained displaced depiction of confidence intervals within the Bland-Altman plots in Figs. 1 and 2, which have been adjusted. Additionally, the corresponding titles of the plots were interchanged in the initial version of the manuscript and have been corrected accordingly. The updated figures (Figs. 1 and 2) are published in this corrected article.

**Fig. 1:**
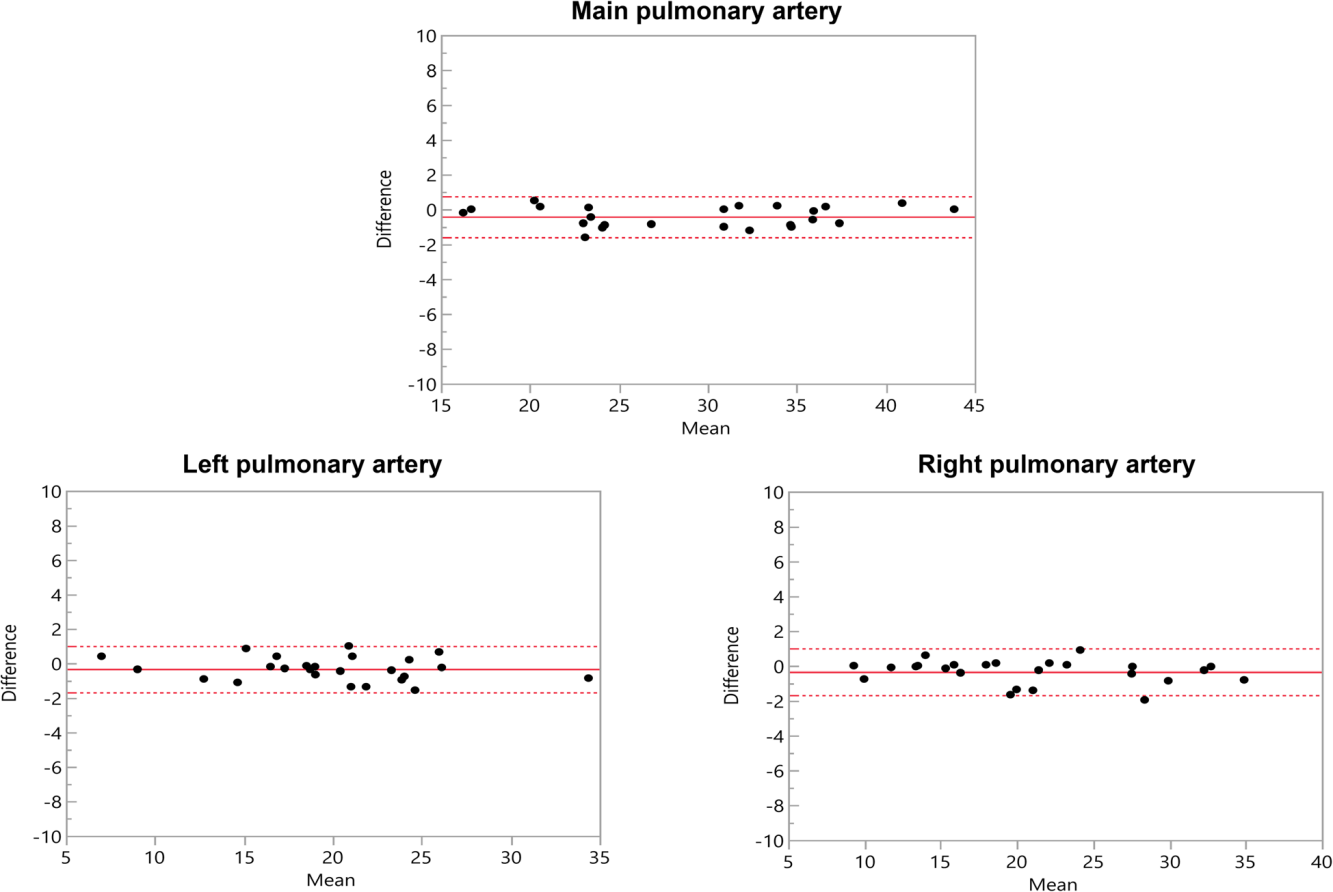
Bland–Altman comparison of the measured diameters of the pulmonary arteries assessed by modified REACT-non-CE-MRA and 4D CE-MRA. The middle line indicates the mean bias of the diameter measurements whereas the dotted lines represent the 95% confidence interval. Values are given in mm

**Fig. 2:**
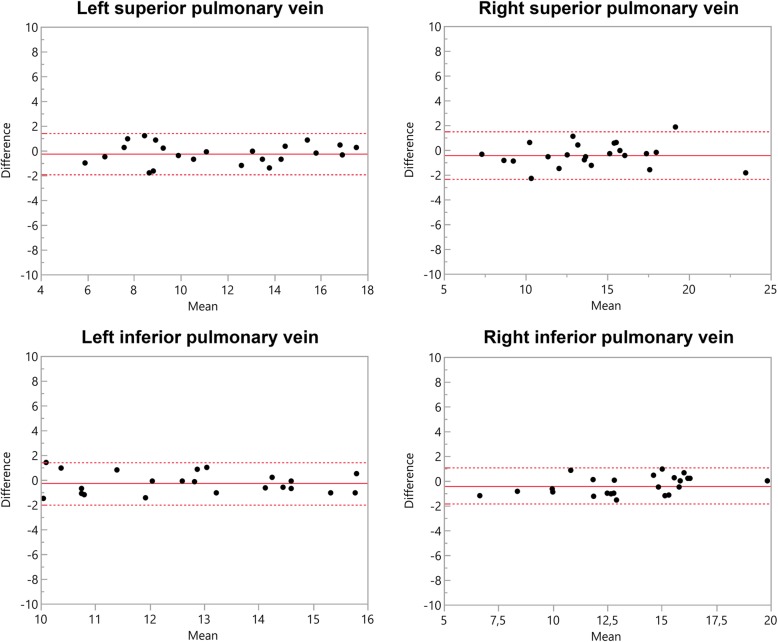
Bland–Altman comparison of the measured diameters of the pulmonary veins assessed by modified REACT-non-CE-MRA and 4D CE-MRA. The middle line indicates the mean bias of the diameter measurements whereas the dotted lines represent the 95% confidence interval. Values are given in mm
